# Closeness and separation in neonatal intensive care

**DOI:** 10.1111/j.1651-2227.2012.02787.x

**Published:** 2012-10

**Authors:** Renée Flacking, Liisa Lehtonen, Gill Thomson, Anna Axelin, Sari Ahlqvist, Victoria Hall Moran, Uwe Ewald, Fiona Dykes, the SCENE group

**Affiliations:** 1 Department of Women’s and Children’s Health, Uppsala UniversityUppsala, Sweden; 2 School of Health and Social Studies, Dalarna UniversityFalun, Sweden; 3 Department of Pediatrics, Turku University Hospital, and Turku UniversityTurku, Finland; 4 Maternal and Infant Nutrition and Nurture Unit (MAINN), School of Health, University of Central LancashirePreston, Lancashire, UK; 5 Department of Family Health Care Nursing, University of CaliforniaSan Francisco, CA, US; 6 Department of Nursing Science, University of TurkuTurku, Finland; 7 Department of Psychology, Turku UniversityTurku, Finland

**Keywords:** Family, Infant behaviour, Intensive care, Neonatal, Parents, Preterm birth

## Abstract

In this paper, we highlight the need for acknowledging the importance and impact of both physical and emotional closeness between the preterm infant and parent in the neonatal intensive care unit. Physical closeness refers to being spatially close and emotional closeness to parental feelings of being emotionally connected to the infant (experiencing feelings of love, warmth and affection). Through consideration of the literature in this area, we outline some of the reasons why physical closeness and emotional closeness are crucial to the physical, emotional and social well-being of both the infant and the parent. These include positive effects on infant brain development, parent psychological well-being and on the parent–infant relationship. The influence of the neonatal unit environment and culture on physical and emotional closeness is also discussed.

**Conclusions:**

Culturally sensitive care practices, procedures and the physical environment need to be considered to facilitate parent–infant closeness, such as through early and prolonged skin-to-skin contact, family-centred care, increased visiting hours, family rooms and optimization of the space on the units. Further research is required to explore factors that facilitate both physical and emotional closeness to ensure that parent–infant closeness is a priority within neonatal care.

## Introduction

Physical closeness in a neonatal intensive care unit (NICU) ranges from skin-to-skin contact between parent and infant, to parents being in the unit but not in physical contact with their infant. Emotional closeness describes how parents can experience anything from feelings of strong and consistent love, care, affection and/or connection to emotional disconnection and alienation from their infant. Although ‘physical closeness’ may facilitate ‘emotional closeness’ and *vice versa*, there may be occasions when parents can be physically close but feel emotionally detached, or even physically remote but still feel emotionally connected. In this paper, we highlight the importance and potential impact of both physical and emotional closeness and the deleterious effects of separation between a preterm infant and the parent during neonatal care.

## Brain Development and Long-Term Outcome of the Infant

The brain of a preterm infant is immature and vulnerable and, therefore, preterm infants are at a risk for abnormal brain development and later developmental problems. However, they also have large brain plasticity and potential for injury compensation. A growing body of evidence in both humans and animals suggests that brain development and later development may be influenced by the quality of care given to preterm infants including physical and emotional closeness and parent empowerment. Mother–infant interaction in early postnatal life, or lack of it in case of separation, can mediate variations in offspring phenotype, including emotional and cognitive development, with long-term health consequences. Environmental factors can influence gene expression through epigenetic mechanisms to provide the ‘plasticity’ necessary to respond to variations in environment (1). Term infants born to mothers with high levels of depression and anxiety during the third trimester have been shown to display increased DNA methylation in cord blood cells and increased salivary cortisol in response to stress at 3 months of age (2). Early life separation can alter capacity to regulate responses to stressful events as illustrated in animal studies (3,b4). Furthermore, animal studies show that prolonged or repeated physical separation between parent and newborn alters brain development (5), impairs the ongoing bonding/attachment process and has long-lasting effects on, for example, emotional programming (6,b7). In preterm infants, cortisol levels have been shown to be higher when cared by depressed mothers compared with nondepressed mothers (8), an effect not seen in term infants. In contrast, close physical contact between parent and preterm infant decreases infant’s cortisol levels and pain responses (9) and family-centred care, providing more parent–infant closeness, synchronizes cortisol variation between the preterm infant and mother (10).

**Key notes**

- The evidence suggests many benefits of early parent-preterm infant closeness during hospital care.
- In future, we need to explore facilitating and inhibiting factors to be able to implement strategies supporting closeness.
- Attention should be paid to both architectural structure and organizational culture in the neonatal units to support both the physical and emotional needs of parents and infants.

Skin-to-skin contact, developmental care and other interventions supporting parenting and parental involvement in infant care have been shown to have the potential to enhance neurological and neurobehavioural outcomes of preterm infants (11–b13). Parental closeness can be lead to improved child outcomes by many mechanisms. One mechanism might be improved sleep, which has been associated with skin-to-skin contact in preterm infants (13,14). Second, parent’s participation in pain management may reduce pain in preterm infants and moderate the use of pain medication (15). Third, infant massage with moderate pressure may increase the concentrations of hormones such as brain growth-promoting factor, IGF-1 (16) and oxytocin, potentially having positive effects on the brain functioning and development. Fourth, the presence of a parent may give the preterm infant learning experiences that he/she might not get otherwise, such as interactive communication. Caskey et al. (17) showed that exposure to parent talk in the NICU was a significantly stronger predictor of preterm infant vocalizations than talk from other adults. These mechanisms might underlie the finding that physical contact enhances early neurobehavioural and psychomotor organization (18–b20). In addition, interventions supporting parents in their skills to observe and interpret their infant’s behaviour have been associated with improved cognition years later (21–b22). Such interventions may restore and normalize the parent–infant relationship even after initial separation.

## The Emotional and Social Well-Being of the Parent

A preterm birth has been associated with poor psychological functioning in mothers and fathers, and in more negative parental interactive behaviours with their infants. Higher prevalence of depression in parents of preterm infants compared with those of full-term infants may be explained by interrupted psychological processes during pregnancy, a stressful birth, concern for their infant’s well-being and NICU experiences (23–b24). However, it is plausible that separation from the infant is one mechanism that increases the risk of parental depression. Early physical separation from the infant within 24 h of birth is related to an increase in parents’ NICU-related stress (25). Furthermore, prolonged physical separation between mothers and infants is also known to cause maternal stress, anxiety and depression (26). Isolation between parents and infants, often attributed to the complex technological support crucial for the infant’s viability, can place immense strain on parents leading to parents feeling less confident and more alienated from their infants and incompetent in the parental role (24, 27). Whilst poor and restricted staff–parent interaction and communication can exacerbate parents’ sense of isolation from their preterm infants, it has also been suggested that parents’ negative emotions and experiences associated with prematurity or infant illness have led them to withdraw physically and emotionally, thereby handing over the care of their infants to staff (27–b28). Emerging evidence suggests that care practices supporting physical and emotional closeness between the parent-preterm infant decrease the prevalence of maternal depression similar to levels reported in mothers of full-term infants (29–b30). Furthermore, psychological well-being of the parents of preterm infants has a long-term impact in terms on later child behaviour (31).

## The Secure Parent–Infant Dyad

Parental attachment to the infant, also called psychological bonding, begins and is strengthened throughout pregnancy (32). After birth, close physical contact with the newborn is crucial for this bond to develop into a secure attachment relationship between parents and their infants (33). Research has shown that newborn infants have the capacity to exhibit sensory awareness, express emotions and share feelings (34). These abilities enable infants to engage in very complex early social relationships with their parents, which form the basis for the evolving parent–infant relationship and attachment (7, 35).

A recent meta-analysis (36) was undertaken to explore mother–infant interactions and relationships within the preterm and full-term populations. The results revealed that, during first 6-month post birth, mothers of preterm infants demonstrated less positive interaction behaviours with their infants than mothers of term infants. However, this review also identified how mothers of preterm infants were as likely to form secure attachments as full-term infants and their mothers at 1 year of infant’s corrected age. Whilst this review focused on the whole preterm population, research targeting infants requiring intensive supervision and surveillance, and hence early and long periods of separation from their parents, identified different results. A qualitative study of attachment revealed that mothers of very low birth-weight infants who experienced prolonged separation displayed more negative attachment behaviours compared with mothers of healthy full-term or preterm infants (37). It has also been suggested that the lack of physical contact between the mother and infant after birth is associated with later emotional problems in preterm infants (38). Studies undertaken with fathers and their preterm infants have also identified an association between early contact and feelings of emotional closeness (39) and more positive interactions at discharge (14). Goulet et al. (40) described how physical closeness and emotional closeness (through vocalizations, visual contact, touch and other sensorimotor interactions) are crucial to the establishment of the parent–infant relationship. Whilst close contact facilitates the development of positive parent–infant relationships, it can also enhance the parent’s confidence and capabilities in providing care for their newborn. Further studies have concluded that maternal sensitivity in mothers with preterm infants is less optimal when compared with full-term controls (41–b42). Research has identified how mothers of preterm infants may be more controlling, actively engaged and/or intrusive with their infants, perhaps compensating for guilt/shame for not having been the caregiver they wanted to be during hospitalization or for preterm infants’ inactive interaction (28,41,43). These findings emphasize that close physical contact may be important and powerful for the formation of secure and healthy attachment relationships.

Feeding is one of the most prominent care-giving activities in a NICU, in which the transition from tube feeding to breastfeeding is complicated by the degree of prematurity, emotional exhaustion, mother–infant separation, institutional authority and by a view of breastfeeding as a productive process, thereby preventing mothers’ experiences of breastfeeding as reciprocal and ‘successful’(28,44). Early physical closeness and breastfeeding have been described by many mothers as ‘steps towards normality’, nurturing the intimate mother–infant interplay (28,44). Skin-to-skin contact has been highlighted as an important intervention to promote breastfeeding, in which oxytocin release is suggested to be an important mediator for the effects of close physical contact on breastfeeding (45). Moreover, long periods of mother–infant skin-to-skin contact are regarded as an effective way to empower mothers to become familiar with their infants, strengthen their mothering at their own pace and increase feelings of parental competence (46).

## The NICU Architecture Facilitating Closeness

Evidence-based architecture has provided research on the benefits of different options concerning the physical structure of a neonatal unit. There is a trend towards single family room design when building new units (47), which started at step-down units (e.g. at Rainbow Babies’ and Children’s Hospital, Cleveland, Ohio, opened in 1997), spread to intensive care units (e.g. at Blank Children’s Hospital, Des Moines, Iowa, opened in 2001) and has been increasingly replacing traditional open-bay design units worldwide. This architectural structure provides the family with an opportunity to be with their child in the neonatal intensive care unit day and night providing facilities for parents’ basic needs including the need for privacy. This design has been suggested to be associated with a lower rate of hospital-acquired infections, similar to single patient rooms in adult intensive care (48), earlier full enteral nutrition, higher breastfeeding rates and a more soothing environment with, for example, lower ambient sound levels (49). As this design has been shown to reduce the length of stay in hospital significantly, for example, by 10 days in preterm infant below 30 weeks of gestation in a Swedish study (50), it shortens the time of separation for the infant from the home and family. Parents have reported that they felt that a single family room design in a NICU facilitated their presence with their infant (51), but the increase in parent–infant closeness gained by a single family room model during hospital care is not well documented in scientific literature.

There are ways, even in traditional open-bay units, to increase parent’s facilities to be close to the infant, for example, by providing comfortable chairs and/or beds for them. Parents’ presence can also be promoted by improving privacy by visual separation and ear phones when other families’ issues are discussed in the same room. There is a very large variation between the neonatal units as to the extent to which they offer such facilities. One survey reported that reclining chairs were offered for parents in 11–100% of units and beds in 0–100% of units in different European countries (52). Based on these data, it can be concluded that there is likely to be a great variety in the time parents spend with their infants in different units/countries.

## The NICU Culture Facilitating Closeness

Parallel to structural changes, there is an ongoing change in the care culture in neonatal units to support parenting in the context of neonatal intensive care. Even though there has been a change in the attitude in neonatal care towards a more family-centred approach, there is still a medical and technical focus and there seems to be a gap between care policies/practices and evidence from family and infant research (53). Furthermore, parents’ visits to their infants on NICU are still limited in many European countries and many units do not allow parents present during medical ward rounds, nursing shift handovers and ‘quiet periods’(52). Whilst very few studies have looked at parents’ visiting patterns, Franck & Spencer (54) showed most mothers visited the NICU daily with a mean length of 3 h. Only a third of the fathers visited on a daily basis and their visits were shorter. Infrequent maternal visits have been identified as a risk factor for later psychological development in preterm infants (55). However, some parents have fewer means to be with their preterm infant during the hospital stay. Older siblings, long travelling distance to hospital or short parental leave limit the parents’ opportunities to be present at NICU. In such cases, modern technology could be utilized to support parent–infant contact. Web camera connection for parents has been used as a method for ‘virtual visitation’ of a neonatal unit (56).

To facilitate physical contact between parents and their infants, neonatal unit staff need to welcome parents’ participation in the care but also guide parents when adapting parental touch into daily care, as touch may induce stress in very ill infants (57). In a genuinely family-centred culture, institutional powers are limited and the role of the staff is altered from ‘doing’ and supervising to becoming a resource and a facilitator. Hence, when family-centred care is implemented in a professional-centred caring culture, this can highlight issues about control and power or unclear responsibilities (58), which pose a considerable challenge for the current care culture. Thus, an important aspect of organizational culture centres upon the ways in which staff are facilitated to build relationships with parents. As parent–infant bonding is a primary goal, successful transition requires education and feedback to the staff as particular demands on staff and care will follow (46). Different interventions to increase parental involvement and empowerment during the neonatal care have already been performed and reported on: parents have been involved in pain management by holding the preterm infant (15); parents have been supported in observing and interpreting their infants behaviour (29); parents have been encouraged to give extended skin-to-skin care (59). Supporting parents’ abilities to interpret their infant and supporting their empowerment has significantly shortened the length of hospitalization (29), decreasing separation of the infants from family and home. Although many short- and long-term benefits have been shown after these types of interventions, there is a lack of research on how these interventions change care culture and affect parent–infant closeness during neonatal care.

Large and systematic differences related to cultural and contextual issues in neonatal units, such as parental involvement, implementation of family-centred care and staff practices, might influence differences shown in breastfeeding rates, maternal depression, and short- and long-term outcomes of the children (60). There is a need to evaluate differences in parent–infant closeness/separation between the units and structural, cultural and socio-economic factors affecting the differences. These factors could be evaluated using qualitative and quantitative techniques including ethnography.

## Conclusion

There is increasing evidence supporting the benefits of early parent–infant closeness during hospital care of preterm infants. Both physical and emotional parent–infant closeness should be facilitated in neonatal units taking into account the socio-economic, political and cultural variations in different countries. To better understand the issues, we need to explore what facilitates and inhibits closeness and consider implementing strategies that enable closeness. These strategies include optimizing the spatial configuration of the neonatal unit; providing chairs/beds/privacy within the given architectural design; developing a nurturing unit culture by removing all restrictions with regard to parents being on the unit and including them as empowered players in the care effort. The most important consideration is paying attention to developing an organizational culture that supports the formation of parent–infant relationships, that is, the physical and emotional needs of parents and infants.
